# Directed brain connectivity biomarkers of healthy aging and Parkinson’s disease staging

**DOI:** 10.3389/fnagi.2025.1698600

**Published:** 2025-10-28

**Authors:** Tauqeer Anjum, Ali Seyfizadeh, Hao Ding, Tahir Mahmood, Rüdiger Pryss, Jens Volkmann, Dumitru Ciolac, Muthuraman Muthuraman

**Affiliations:** ^1^Department of Neurology, University Hospital Würzburg, Würzburg, Germany; ^2^School of Natural Sciences (SNS), National University of Sciences & Technology (NUST), Islamabad, Pakistan; ^3^Institute of Clinical Epidemiology and Biometry, University of Würzburg, Würzburg, Germany; ^4^Institute of Medical Data Science, University Hospital Würzburg, Würzburg, Germany; ^5^Nicolae Testemitanu State University of Medicine and Pharmacy, Chisinau, Moldova; ^6^Institute of Computer Science, University of Augsburg, Augsburg, Germany

**Keywords:** fMRI, directed connectivity, Parkinson’s disease, prodromal PD, temporal partial directed coherence

## Abstract

**Introduction:**

The propagation of neural signals across various brain regions requires us to understand directional connectivity in functional magnetic resonance imaging (fMRI) data. We employ temporal partial directed coherence (TPDC), a data driven method to explore directional connectivity in young and old healthy individuals, manifest PD and prodromal PD patients. TPDC provides comprehensive analysis of neural signal propagation compared to traditional methods like Dynamic Causal Modeling, Granger Causality and Transfer Entropy.

**Methods:**

We analyzed preprocessed fMRI data from the young and old groups of healthy individuals and PD patients at different disease stages. Time series were extracted by reducing the voxel data (by averaging) into 7 networks of the Yeo-atlas. TPDC was applied in the frequency range of 0.009–0.08 Hz. Statistical significance of connections was determined via bootstrapping, followed by thresholding using permutation testing. Finally, machine learning classifiers were trained to distinguish prodromal PD from PD patients.

**Results:**

In young healthy individuals, the somatomotor network regulates control and attention systems, indicating cognitive and motor flexibility. Older healthy controls show lack of significant connections from control to somatomotor networks, suggesting a cognitive decline related to age. The somatomotor network becomes secluded in the prodromal PD patients. A compensatory mechanism is visible in groups of PD patients. Additionally, machine learning classifiers achieved high accuracy in distinguishing between prodromal and PD groups based on directed connectivity patterns.

**Conclusion:**

The study highlights the gradual loss of the significant directed causal connections between the control and motor networks in different stages of PD. The governing influence of control network over the motor and attentional networks diminishes, leading to the isolation of the somatomotor network. The ability of TPDC-derived features to distinguish prodromal from Parkinson’s patients underscores its value for identifying potential biomarkers of disease onset and progression.

## Introduction

Parkinson’s disease (PD) is a progressive neurodegenerative disorder affecting motor and non-motor (cognitive, autonomic, affective, behavioral) functions in part due to pathological disruption of fine-tuned neural networks. Patients with PD exhibit an intricate pattern of functional connectivity alterations within the major brain networks as evidenced by electroencephalography and neuroimaging studies (Leviashvili et al., 2022). Particularly, reduced connectivity within the central executive and dorsal attention networks, as well as increased connectivity within the ventral attention network were observed (Leviashvili et al., 2022). Moreover, disruptions in interhemispheric connectivity may substantially interfere with the execution of complex movements in patients with PD (Bange et al., 2022). Depicting connectivity patterns in different stages of PD might provide additional insights into the pathophysiology of the disease and refine the existing treatment approaches for these patients. Therapeutic interventions targeting abnormal oscillatory activity within brain networks, e.g., deep brain stimulation, were shown to modulate the deficient connectivity patterns and provide clinical benefits (Muthuraman et al., 2018).

Functional connectivity is characterized by statistical dependence of activation patterns across different brain regions, resulting from both indirect and direct neural interactions (Firston, 2011). One previous research laid the foundational work by assessing functional connectivity in the human brain using functional magnetic resonance imaging (fMRI) data (Rogers et al., 2007). More recently, resting-state fMRI data was used to infer regional functional connections (Mansour et al., 2023). A multivariate measure, total correlation was utilized to explore functional brain connectivity (Li et al., 2022). The human functional connectome across the lifespan using both the structural and functional MRI data has previously been explored (Sun et al., 2023). While the functional connectivity captures the statistical dependencies between different brain regions, it lacks information about the direction of these interactions. Our study focuses on directional connectivity, which we believe would be particularly useful for patients’ classification.

Directed connectivity based on fMRI data is traditionally assessed using methods like dynamic causal modeling (DCM) (Bajaj et al., 2016), granger causality (GC) (Kaminski et al., 2001) and transfer entropy (TE) (Duan et al., 2013). These methods provide insights into the neural signal propagation across different brain regions, thereby granting a non-invasive access in deciphering the intricate interactions across brain networks. Dynamic causal modeling is a hypothesis-driven method that models causal interactions between different brain regions taking into account hemodynamic and neuronal responses (Friston et al., 2003). On the other hand, TE is a data-driven approach that offers a thorough analysis of neural data in the time domain by capturing nonlinear interactions and measuring the directed exchange of information between the time series (Schreiber, 2000). Temporal partial directed coherence (TPDC) analysis is purely a data-driven analysis and an extension of the partial directed coherence, a technique designed to analyze multivariate time series data and infer causal relationships between various brain regions. Temporal partial directed coherence focuses on both time and frequency domains, allowing for a comprehensive characterization of connectivity patterns at all time points and frequency bands (Baccalá and Sameshima, 2001). One of the major advantages of TPDC analysis is its ability to uncover the dynamic patterns of brain connectivity that engage different functional brain networks.

By employing the TPDC analysis, we aimed to establish the existing patterns of directed connectivity in three different cohorts - healthy individuals, patients with manifest PD and patients with prodromal PD. Connectivity patterns emergent from this study might be used as open-source reference patterns in future works studying connectivity alterations in different patient populations to disentangle healthy aging from disease-specific connectomes in neurodegenerative disorders.

## Materials and methods

### Subjects

This study included the preprocessed fMRI data from 1,000 young healthy participants (male to female ratio 1:1), publicly available at Harvard Dataverse as “GSP1000 Preprocessed Connectome” (Cohen et al., 2020). For each subject, the average blood oxygen level-dependent (BOLD) time series from brain networks (Yeo et al., 2011), were extracted using a custom Python script that utilizes “nilearn” python library.^1^ This study also included data collected for a group of manifest PD patients (*n* = 435), prodromal PD patients (*n* = 325) and old healthy controls (*n* = 29) from the openly available PPMI (Parkinson’s Progression Markers Initiative) dataset.^2^ Clinical scores like behavioral inhibition score, Barratt impulsivity score and conscientiousness score were collected for young healthy controls, whereas sum of motor exam score (NP3 Total) and Hoehn and Yahr (H&Y) stage values were collected for the prodromal PD and manifest PD patients from PPMI. Demographical and clinical details of the subject and patient groups are given in Table 1.

**Table 1:** Demographical and clinical characteristics of patients and healthy controls.

	Prodromal PD patients, PPMI	PD patients, PPMI	Young healthy controls, GSP1000	Old healthy controls, PPMI
*N*	325	435	1,000	29
Male/female	185/140	299/136	500/500	22/7
Age, mean ± SD	66.7 ± 6.5	63.4 ± 9.7	21.4 ± 2.87	61.8 ± 5.1
NP3 total, mean ± SD	3.56 ± 5.68	22.16 ± 10.47	–	–
H&Y scale median (range)	0 (0–3)	2 (1–4)	–	–
Behavioral inhibition score (BIS) median (range)	–	–	21 (7–28)	–
Barratt Impulsivity score median (range)	–	–	62 (30–120)	–
Conscientiousness score median (range)	–	–	32 (0–48)	–

GSP, Genomics Superstruct Project; H&Y, Hoehn and Yahr scale; NP3 total, sum of motor exam scores; PPMI, Parkinson’s Progression Markers Initiative; SD, standard deviation.

The NP3 total is a detailed measure of motor symptoms in PD. It includes scores from multiple individual motor tests such as tremor, rigidity etc. conducted during a clinical motor exam. A higher NP3 score indicates more severe motor impairment. Hoehn and Yahr (H&Y) scale classifies the overall severity of PD. BIS is the behavioral inhibition score on the behavioral inhibition and behavioral activation (BISBAS) scale. Barratt impulsivity score is the total score on the Barratt impulsivity scale (Patton et al., 1995). The conscientiousness score signifies the NEO five-factor model of personality.

**Figure 1:**
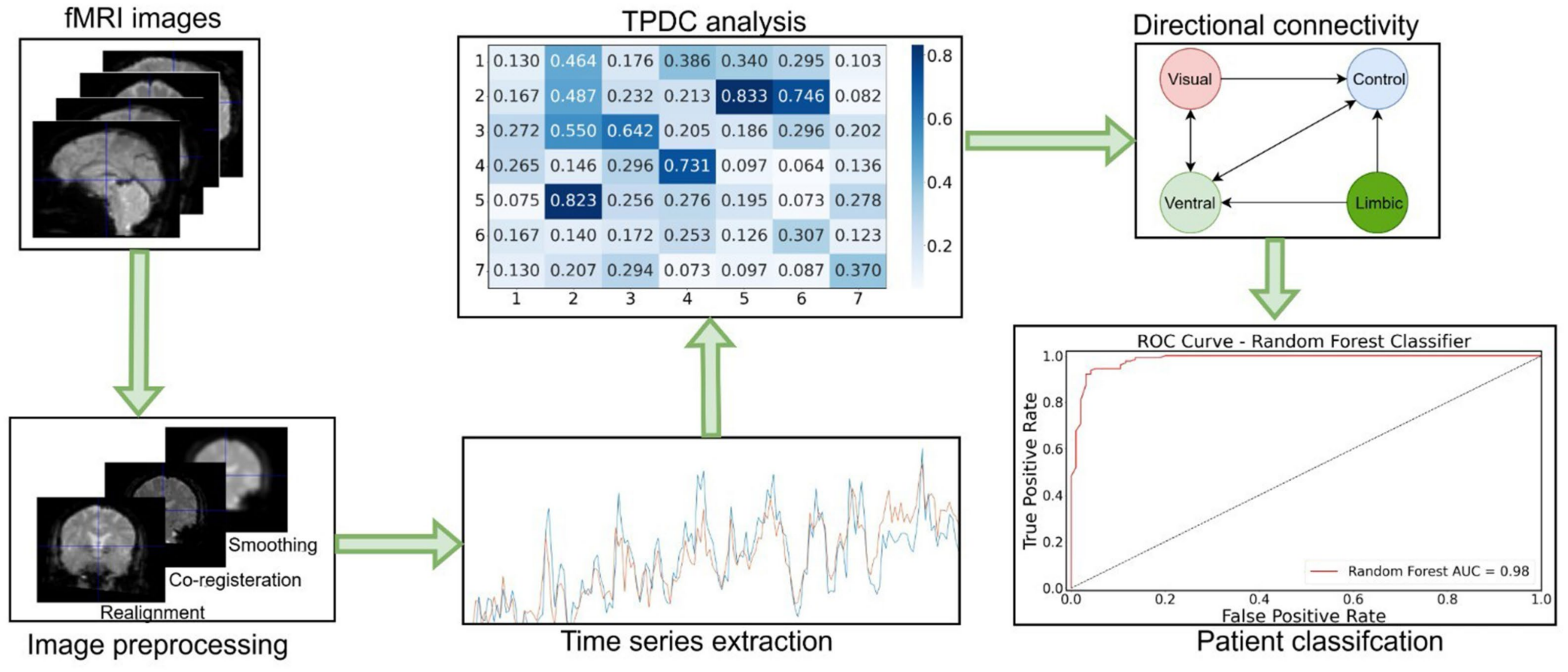
Workflow diagram of the fMRI data analysis. First, fMRI images were preprocessed and time series extracted. Then, TPDC analysis was performed to assess the directed connectivity between Yeo-7 networks. Afterwards, significant directed connections were detected by applying the random permutation testing approach. At the end, random forest machine learning algorithm was used to classify the patients based on their individual connectivity values.

The PPMI data was preprocessed using a preprocessing pipeline that included realignment and coregistration followed by smoothing using an 8 mm kernel. The preprocessing of these fMRI images was performed using the SPM-12 Matlab toolbox.^3^ Then, the time series were extracted for the Yeo-7 network parcellation atlas, which includes the following networks: visual, somatomotor, ventral attention (or salience), dorsal attention, frontoparietal control, limbic, and default mode networks. The python library “nilearn”^4^ was used to extract the time series from the Yeo-7 networks (Yeo et al., 2011), based on the custom python script with a spatial resolution of 1 mm. Afterwards, these time series were utilized for the TPDC analysis. Eventually, thresholding was performed to obtain significant connections for each group of subjects from the study. The detail of the applied methodology is shown in Figure 1.

### TPDC analysis

In order to determine the causality between the time series, we used the time-dependent multivariate coefficients. Dual extended Kalman filters can be used as a foundation for the causality estimation (Haykin, 2001; Wan and Nelson, 2001). This functions as TPDC method’s estimation strategy for the time-dependent auto-regressive coefficients. To find the partial directed coherence at each time point, time-dependent multivariate auto-regressive coefficients were computed. TPDC as an extension of partial directed coherence, captures both time and frequency resolved directional interaction in a multivariate time series given a multivariate autoregressive (MVAR) model of order p.


$$X(t)=\sum_{k=1}^{p}A_{k}(t)X(t-k)+E(t)$$

Where X(t) is the vector of fMRI time series across regions, $A_{k}(t)$ are the time varying autoregressive coefficient matrices and E(t) is the residual noise. The model order was fixed at *p* = 1. TPDC is computed as:


$$\pi_{\mathrm{ij}}=\frac{A_{\mathrm{ij}}(f,t)}{\sqrt{\sum_{k=1}^{N}|A_{\mathrm{kj}}(f,t)|2}}$$

Where $A_{\mathrm{ij}}(f,t)$ is the Fourier transform of MVAR coefficients, $\pi_{\mathrm{ij}}(f,t)$ quantifies the normalized directed influence from region *j* to *i* at frequency *f* and time *t*. Normalization is performed to reduce the confounding effect of the indirect connections. We focused on the frequency band in range from 0.009 Hz - 0.08 Hz as it corresponds to the range believed to reflect brain physiology and metabolism (Logothetis and Wandell, 2004; Deco et al., 2011). The TPDC matrix (e.g., 7*7 matrix) was then obtained by averaging all the frequency bins in the frequency band of interest. To determine the model parameters and the system states at any given moment, the two Kalman filters were run in parallel. The states were estimated by one extended Kalman filter, which then feeds the data to another extended Kalman filter, which estimates the model parameters.

The significance level of the estimated TPDC values was determined using a bootstrapping technique (Kaminski et al., 2001). The original time series were divided into smaller, non-overlapping windows, and the order of these windows was randomly changed to create the new time series. Next, using the updated time series as a basis, the TPDC matrix was computed. The shuffling operation was carried out 100 times to calculate the threshold, after which the 99^th^ percentile was determined. The autoregressive coefficients were estimated from the spatially filtered source signals using the open-source MATLAB ARFIT toolbox (Neumaier and Schneider, 2001; Schneider and Neumaier, 2001).

### Directed connectivity analysis

Significant values that were above the significance threshold were replaced by 1’s and those below the significance threshold were replaced by 0’s. In this manner, the causal relationships between any two regions were represented in a significance matrix of ones and zeros. We then employed permutation testing to assess the statistical significance of the observed connectivity matrices. To generate the null distributions for each connection, we performed 1,000 random permutations. In each permutation, the matrices were flattened and randomly shuffled, only preserving the marginal totals. The shuffled data were then reshaped and aggregated across subjects, to form a frequency matrix. The 99th percentile of the resulting null distribution was used as a significance threshold. Connection values above this threshold were considered statistically significant, indicating a significant directed connection and the values below the threshold were considered insignificant, implying in the absence of significant directional connectivity.

Afterwards, the obtained individual connectivity matrices were used to find group level directed connections. A data-driven, Moran’s permutation testing approach was applied to evaluate the spatial autocorrelation and identify the statistically significant inter-network connections (Moran, 1950; Zelesky et al., 2010). This method allows to compare the identified connectivity patterns to a null model derived from the permuted datasets, offering data-driven statistically significant directed connections. In more detail, the algorithm runs in following steps. For each inter-network pair, the observed connections’ frequency across the groups is computed by summing up the values into a three-dimensional binary connectivity matrix. To generate the null distribution for each connection, 1,000 permutations of the individual matrices were performed. Each individual matrix was flattened and shuffled randomly. The permuted matrices were then reshaped and aggregated to generate a permuted frequency matrix. For each connection, 99th percentile value from its null distribution was used as a significance threshold. Connections with the values exceeding that threshold were represented as a significant directed connection, while the values below the threshold were regarded as lacking significant directed connectivity.

### Classification analysis

We also used machine learning algorithm able of classifying individual patients as either prodromal PD or manifest PD patients based on their individual directed connections. The individual directed connectivity matrices (7*7) for the patient groups were therefore flattened into a one-dimensional feature vector for each subject. The python library “scikit-learn” was used for the classification analysis. A random forest classifier with 100 decision trees was trained using the features matrix to distinguish between the two patient groups.^5^ The data was split into training and testing sets (70:30 stratifies ratio) to maintain class distribution. Performance of the model was evaluated using metrics such as accuracy, precision, recall and the F1-score. To further ensure robustness and assess potential overfitting, we additionally performed a stratified 10-fold cross-validation. The average accuracy and standard deviation across the folds were computed to eliminate the risks of overfitting (Supplementary Table S1).

### Statistical analysis

We conducted an analysis of variance (ANOVA) test to examine the differences in age across the four groups in our study, using MS EXCEL’s (Microsoft 365) built-in ANOVA: Single Factor tool available in the Data Analysis Toolpak. To account for multiple comparisons, *post hoc* tests were performed using Bonferroni correction. The differences in TPDC values for causal connections among the sample of groups of controls and patients were tested using a non-parametric Friedman test (*n* = 29, *p* = 0.05). To examine the gender (male/female) distribution between the four groups, we performed Chi-square test of independence.

Associations between the TPDC values and clinical scores in old healthy controls, patients with prodromal PD and manifest PD patients were found. In contrast, associations between the TPDC values and the behavioral scores like behavioral inhibition score, conscientiousness score, barratt impulsivity score, were assessed for young healthy controls. Spearman’s rank correlation was used for H&Y stage, while Pearson’s correlation was used for continuous normally distributed variables, using custom Python script (scipy pearsonr, spearmanr). The differences in the TPDC values were evaluated using linear regression analysis with a significance level of 0.05.

## Results

### Subject characteristics

The age of subjects differed between the groups (*F*(3,1785) = 7732.19, *p* < 0.001). Particularly, prodromal PD patients were older compared to manifest PD patients and old healthy controls (*post-hoc p* < 0.001). However, no significant differences were found between manifest PD patients and old healthy controls (post-hoc *p* = 0.392). A Chi-square test of independence showed a significant difference in gender distribution across the groups (*χ*^2^(3) = 48.02, *p* < 0.0001) - balanced gender distribution in the young healthy control group, while the older healthy and patient groups have a higher proportion of male participants, which aligns with the known male predominance in Parkinson’s disease prevalence.

### Directed network connectivity

By applying the TPDC analysis followed by Moran’s permutation testing approach, we examined the significant directed connections among the following seven major brain networks of the Yeo-7 atlas, namely the ventral attention (VAN), dorsal attention (DAN), limbic (LIN), frontoparietal control (FPN), somatomotor (SMN), visual (VIN), and default mode (DMN) networks. These connections are represented in the adjacency matrices, where ‘1’ denotes a directed edge connecting any two brain networks. For each group, we obtained separate connectivity matrices, representing significant directed causal connections.

**Figure 2:**
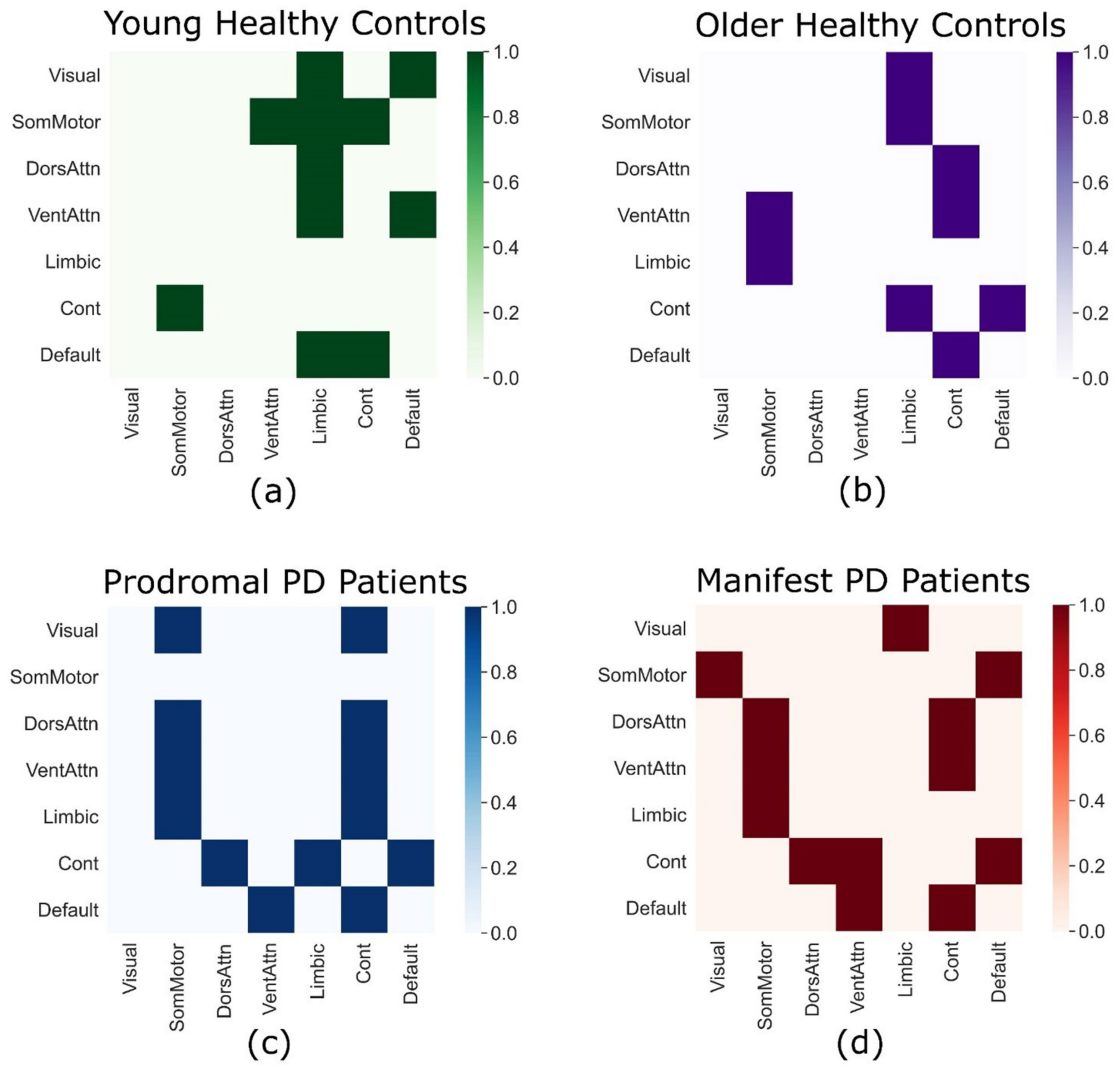
Directed connectivity matrices. Each connectivity matrix displays significant directed connections between the seven major brain networks according to the Yeo-7 atlas in young healthy controls from the GSP-1000 dataset **(a)**, old healthy controls from the PPMI dataset **(b)**, prodromal PD patients from the PPMI dataset **(c)**, and manifest PD patients from the PPMI dataset **(d)**.

In young healthy subjects, significant bidirectional connections between the frontoparietal control and somatomotor networks were observed. These connections were absent in old healthy controls and both PD patient groups. However, compensatory connections between the dorsal and ventral attention networks and the frontoparietal control network were detectable in old healthy controls, prodromal PD and manifest PD patients. The attention networks (Dorsal and ventral) have directed connections to the somatomotor network in prodromal and manifest PD patients which are absent in case of young healthy individuals. The older healthy controls have directed connections from ventral attention and limbic networks to the somatomotor network, which are absent in the young healthy individuals. In addition to that, there is a directed connection from control network to the default network in older healthy individuals and patient groups which is absent in the young healthy individuals. The details of all the connections are available in Figure 2.

By using ordinary least square regression through the *t*-test (*p*-values) and coefficient of determination (*r*^2^), significant differences in TPDC values across the four groups were observed. When comparing young healthy controls and prodromal PD patients, significant differences in TPDC values were found for a sample of connections (*n* = 29), between the dorsal attention network and the limbic network (*p* = 0.047, *r*^2^ = 0.138). While comparing young healthy controls and manifest PD patients, significant differences in TPDC values for the connections between the visual network and the somatomotor network (*p* = 0.027, *r*^2^ = 0.167), between the somatomotor network and the limbic network (*p* = 0.046, *r*^2^ = 0.14), between the ventral attention network and the dorsal attention network (*p* = 0.011, *r*^2^ = 0.215) and between the ventral attention network and the frontoparietal control network (*p* = 0.003, *r*^2^ = 0.28) were detected.

When comparing the TPDC values between the old healthy controls and prodromal PD patients, significant differences in the connections between the visual network and the limbic network (*p* = 0.002, *r*^2^ = 0.302) and between the dorsal attention network and the frontoparietal control network (*p* = 0.048, *r*^2^ = 0.138) were identified. When comparing old healthy controls to age-matched manifest PD patients, significant TPDC differences were observed for the connections between the dorsal attention network and the ventral attention network (*p* = 0.019, *r*^2^ = 0.187), between both the dorsal and ventral attention networks and the limbic network (*p* = 0.036, *r*^2^ = 0.153 and *p* = 0.019, *r*^2^ = 0.187, respectively), and between the frontoparietal control network and the visual network (*p* = 0.016, *r*^2^ = 0.196). No significant differences in TPDC values were found either between young and old healthy control groups, or between prodromal PD and manifest PD patient groups for any connections.

### Patient classification

**Figure 3:**
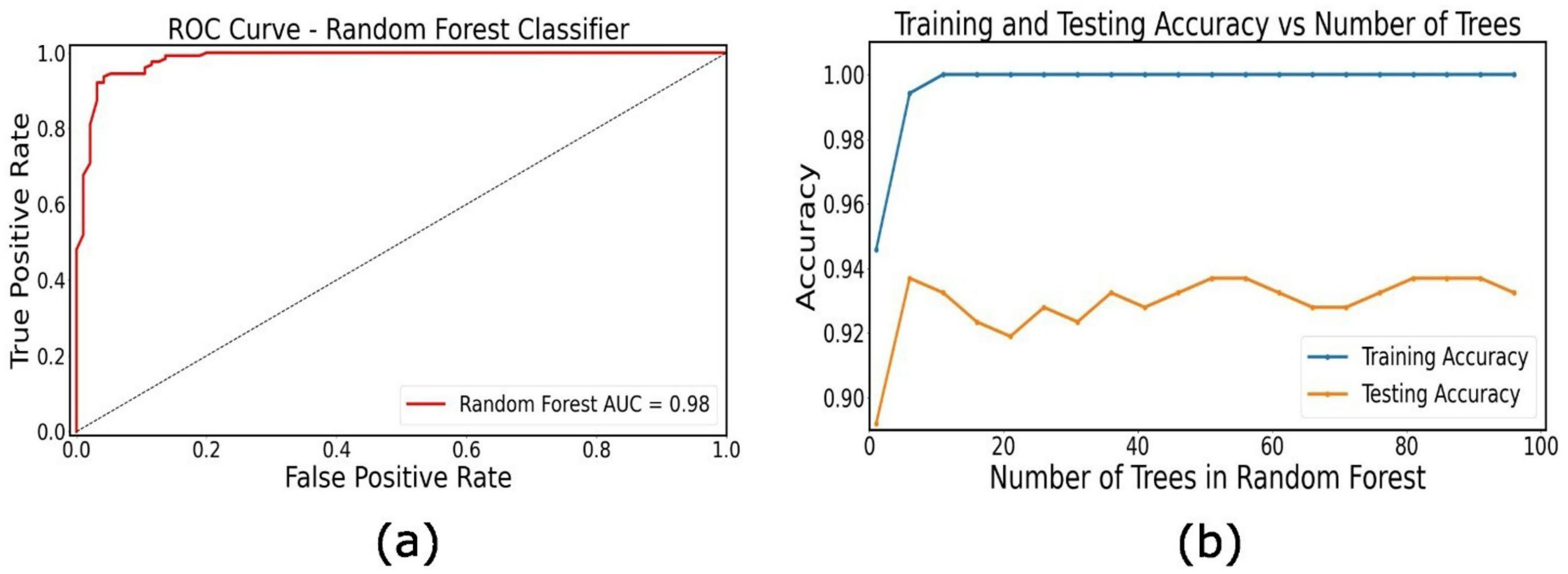
Classification of PD patients. **(a)** Receiver operating characteristic (ROC) curve for the random forest classifier distinguishing between prodromal PD and manifest PD patients based on individual directional connectivity features. **(b)** Training and testing accuracy as a function of the number of trees in the Random Forest model.

The random forest classifier showed high performance in distinguishing between the two PD patient groups. Our model achieved an overall accuracy of 93.24% with precision of 0.95, recall of 0.88, F1 score of 0.92 in classifying the prodromal PD patients, and a precision of 0.92, recall of 0.97 and F1-score of 0.94 for classifying manifest PD patients (Figure 3). To further validate the classification performance, stratified 10-fold cross-validation was performed, yielding a mean accuracy of 94.0% ± 2.5%, consistent with the initial train–test split results (Supplementary Table S1). These results indicate that our model can accurately identify these patients based on their individual directed connectivity patterns and can distinguish between prodromal PD and manifest PD patients. In addition to that, feature importance analysis was also performed to identify top 5 important features to classify PD staging (Supplementary Table S2). These findings also suggest that the directed connectivity patterns combined with machine learning methods, can accurately classify PD at different stages.

### Clinical correlations

**Figure 4:**
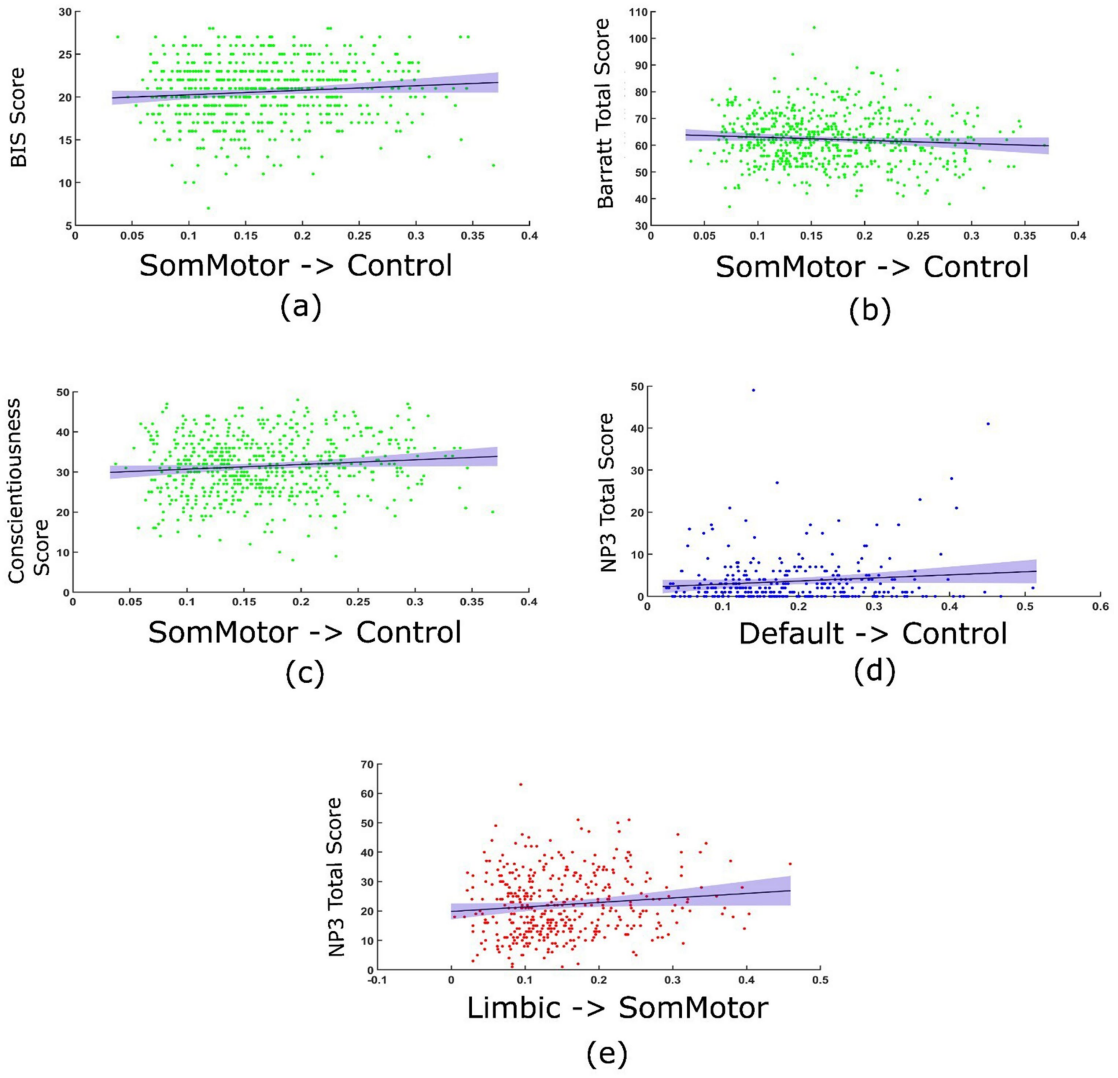
Correlation between the TPDC values and clinical scores. In young healthy controls, TPDC values from the somatomotor to frontoparietal control networks correlated with behavioral inhibition score (BIS) **(a)**, Barrat impulsivity scale **(b)** and conscientiousness score on the NEO five factor model of personality **(c)**. In prodromal PD patients, TPDC value from the default mode to frontoparietal control network correlated with sum of motor exam scores (NP3 total) **(d)**. In manifest PD patients, TPDC values from limbic to somatomotor networks correlated with sum of motor exam scores (NP3 total) **(e)**.

The TPDC values of the connections identified in young healthy controls displayed significant correlations with the clinical scores. Specifically, positive (Pearson’s) correlations between the TPDC values for the connections between the somatomotor network and the frontoparietal control network and the behavioral inhibition scale score [*r* (1000) = 0.094, *p* = 0.019], Barrat impulsivity score [*r* (1000) = −0.0823, *p* = 0.042] and the conscientiousness score [*r* (1000) = 0.105, *p* = 0.009] were detected (Figure 4). Prodromal PD patients showed positive correlations between the TPDC values of the connections between the default mode and frontoparietal control network and the sum of motor exam scores (NP3 total) [*r* (325) = 0.015, *p* = 0.032]. In manifest PD patients, there was a positive correlation between the TPDC values for the connections from the limbic to the somatomotor network and the sum of motor exam scores (NP3 total) [*r* (435) = 0.013, *p* = 0.0176], indicating increased directed connectivity with the progression of motor impairment as seen in Figure 4.

Although correlations between some TPDC values and clinical scores are statistically significant, the effect sizes are very small (all < 0.02). After applying Bonferroni correction to account for multiple comparisons, correlations in manifest PD and prodromal PD patients remain significant, however, for young healthy controls they do not remain statistically significant, which is consistent with their small effect sizes. Hence, the correlations in the healthy and patient groups explained only a negligible proportion of variance, showing that associations likely reflect subtle rather than robust relationships between connectivity and clinical measures.

## Discussion

In this study, we investigated how the directed causal connectivity patterns change across the lifespan and during the progression of PD, especially focusing on the transition of prodromal PD to manifest PD stage. We have identified age and disease related alterations in the directed interactions among important brain networks. In addition to that, we showed that these connectivity values correlate with the clinical and motor scores. Our findings would be helpful in providing important insights into the neural basis and early diagnosis of PD.

### Directed network connectivity

The young healthy controls presented a pattern of significant directed connectivity. Directed connections from the frontoparietal control network to the somatomotor network and from the default mode network to the frontoparietal control network, showed strong top-down regulatory influences, which are essential for healthy brain functioning in young adults. The directed connectivity from the frontoparietal control network to the somatomotor network suggests that these regions play an active role in mediating movement, attention, and cognition (Leisman et al., 2016) in a consistent manner as with the cognitive flexibility and motor control normally observed in young individuals.

Old healthy controls showed a reduction in directed causal connections between different networks. For example, the frontoparietal control network did not exhibit significant connections with the somatomotor network, which was prominent in young healthy individuals, likely due to age-related changes. The somatomotor network had fewer efferent connections that may indicate an age-related motor decline such as balance and gait deficits, control/coordination deficits and slowing of movements (Seidler et al., 2010). There was a lack of directed connectivity from other networks to the dorsal and ventral attention networks, similar to the lack of structural and functional connectivity decline normally observed with the attention networks in older individuals (Zhang et al., 2024) suggesting a decline in the attention mechanisms related to normal aging brain.

### Prodromal PD patients

The prodromal PD group showed early signs of connectivity disruption, primarily in the somatomotor network. The absence of strong outgoing directed connections from the somatomotor network may be related to motor deficits and could possibly lead to PD. Connections from the ventral attention network to the somatomotor and frontoparietal control networks in prodromal PD patients could reflect some compensatory mechanisms (Passaretti et al., 2024), where the attentional network may try to take over some of the motor and cognitive tasks.

### Parkinson’s disease patients

The PD patient group showed a breakdown in connections especially within the frontoparietal control and somatomotor networks, indicating the motor deficits normally seen in PD. The loss of causal connection from the frontoparietal control network to the somatomotor network indicates a disruption in the influence of motor control, which is a clear sign of motor symptoms in PD and may require increased reliance on the attention-based control (Azulay et al., 2006). We observed this in our study, where dorsal and ventral attention networks are highly active and showed significant directed causal connections to the somatomotor and control networks, exhibiting some compensatory attention processes, partly observed in the prodromal PD patients. However, these efforts may not be enough to suppress the disease-related network disruptions.

In addition, we found causal directed connections from the somatomotor network to the visual network, which were present only in PD patients, suggesting the reliance on sensory inputs from the visual system for the guidance of movements. This aligns well with the clinical observations in PD patients, which show increased dependence on the visual cues during gait and balance tasks (Zhang et al., 2023).

### Age-related network alterations

Young healthy controls showed directed connectivity between the somatomotor and control networks reflecting the intricate integration of higher-order functions and motor planning. This significant directed connectivity was absent in the older individuals (older healthy controls, prodromal PD and PD patients), pointing to possible age-related effects on the control and motor functions (Marek et al., 2015; Jockwitz and Caspers, 2021).

In older individuals and patients, there is a notable absence of significant directed connectivity from the attention networks (both ventral and dorsal) to the limbic network, which is responsible for memory processing and emotional regulations, and it tends to deteriorate with advanced age (Gunbey et al., 2014). However, a compensatory mechanism might come into play in the shape of increased connectivity to other networks. Such connections from the attention networks to the control network in older individuals and PD patients my indicate an increased role of attention networks in the aging brain (Ferreira and Busatto, 2013; Watanabe et al., 2021).

Age-related directed connectivity patterns from the limbic network to the somatomotor network were observed in older healthy controls and PD patients, which were absent in the young healthy controls. This significant connection might show some compensatory adaptation to support the motor function as limbic-motor interface is potentially involved in the modulation of complex functions such as spatial awareness and motor coordination (Rizzo et al., 2018). This connection would highlight the role of the limbic network in mediating motor and cognitive tasks in the aging process.

The relatively small sample size of the old healthy control group (*n* = 29) compared to the other cohorts is a limitation and may reduce the robustness of age-related comparisons. While the present analysis provides valuable preliminary insights, future studies should include larger age-matched control cohorts to strengthen generalizability. In addition to that, sensitivity analyses or weighted statistical approaches could be employed to mitigate potential biases arising from group size imbalances.

### Disease-related network alterations

In prodromal PD and manifest PD patients, we observed directed connections from the control network to the dorsal attention network, which were absent in the young and older healthy controls, suggesting some compensatory mechanisms as attention is controlled by frontoparietal control network in PD patients (Seeley et al., 2007; Tessitore et al., 2012). This adaptation might be an attempt by the brain to counter the decline of the motor system with attentional network taking over to maintain the functional movement.

In the prodromal stage we see that the somatomotor network is secluded with no outgoing connections, signaling the early disruption in the integration of motor system, which is a hallmark of PD pathophysiology (Abbruzzese and Berardelli, 2003), however with the progression of the disease we see a significant directed connectivity to visual and default networks in case of PD patients suggesting an adaptation mechanism where disease takes over and reconfigures itself against the motor decline (De Schipper et al., 2018; Thomas et al., 2023). However, this adaptation might not be enough to compensate for the loss of motor functions, highlighting the need for therapeutic interventions such as deep brain stimulation or cognitive training.

### Disease stage classification

Our study shows that the directed connectivity patterns derived from the resting state fMRI data, using the TPDC analysis, can effectively and accurately distinguish between the prodromal PD and Parkinson’s disease patients. The high accuracy, strong performance metrics across both patient group and the ability of our model to classify individual patients based on unique directed connectivity patterns, shows the relevance of this approach which can be particularly useful in the diagnosis of Parkinson’s disease at an early stage. Our findings suggest that changes in the brain network connectivity patterns can help in tracking disease progression over time.

### Clinical significance

We can suggest that the loss of causal connections between the control and somatomotor networks could serve as an early biomarker for PD diagnosis and disease progression. The use of these directed causal connectivity patterns into a machine learning framework resulted into highly accurate classification of PD patients at different stages. Our findings suggest that combining directed connectivity with clinical scores enhances our understanding of PD pathophysiology and holds great potential for early diagnosis and intervention. In light of previous research, our results point to therapeutic strategies like deep brain stimulation or cognitive training, which might help restore this causal connectivity relation and preserve motor control and cognitive function at the early onset of PD.

## Conclusion

The study highlights the gradual loss of the directed causal connections between the control and motor networks, as a significant feature of PD, starting in the prodromal stage and capping at the full-blown expression of PD. The control network loses the governing influence over the motor network, leading to the isolation of the highly important somatomotor network. These disruptions are quite different from the effects of normal aging as seen in the PPMI-healthy older individuals, where the loss of these connections has less influence. The study also highlights the potential biomarkers for early diagnosis and intervention. Some observed connectivity patterns could possibly be of compensatory mechanisms; however, these inferences are correlational, and alternative explanations, such as network reorganization or disease heterogeneity, cannot be excluded. This study aims to contribute to the understanding of directed connectivity patterns in neurodegenerative diseases such as Parkinson’s disease, highlighting the differences in directed connectivity patterns between healthy individuals and Parkinson’s patients.

## Limitations

The relatively small sample size of the old healthy control group (*n* = 29) compared to the other cohorts is a limitation and may reduce the robustness of age-related comparisons. External validation in independent cohorts in future work is required to confirm the generalizability and clinical relevance of these findings.

## Data Availability

The original contributions presented in the study are included in the article/Supplementary material, further inquiries can be directed to the corresponding author.

## Footnotes

^1^ https://nilearn.github.io/stable/

^2^ https://www.ppmi-info.org/access-data-specimens/download-data

^3^ https://www.fil.ion.ucl.ac.uk/spm/software/spm12/

^4^ https://nilearn.github.io/stable/index.html

^5^ https://scikitlearn.org/stable/modules/generated/sklearn.ensemble.RandomForestClassifier.html
