# Supplementary material for: Directed brain connectivity biomarkers of healthy aging and Parkinson’s disease staging
Source: Front Aging Neurosci. 2025 Oct 28;17:1698600. doi: 10.3389/fnagi.2025.1698600 (PMC12602468; doi:10.3389/fnagi.2025.1698600)
Supplement: Supplementary file 1 [file Table_1.docx]

**Supplementary Materials**

**Cross validation analysis**

Supplementary Table S1.

| **Fold** | **Accuracy With TPDC connectivity data** | **Accuracy with GC connectivity data** |
| --- | --- | --- |
| 1 | 0.932 | 0.932 |
| 2 | 0.946 | 0.919 |
| 3 | 0.905 | 0.930 |
| 4 | 0.959 | 0.905 |
| 5 | 0.932 | 0.919 |
| 6 | 0.986 | 0.931 |
| 7 | 0.905 | 0.918 |
| 8 | 0.946 | 0.960 |
| 9 | 0.959 | 0.910 |
| 10 | 0.932 | 0.905 |

TPDC based Mean accuracy: 0.940 (94.0%) - Standard deviation: ± 0.025 (2.5%)

GC based Mean accuracy: 0.924 (92.4%) - Standard deviation: ± 0.015 (1.5%)

**Feature Importance**

Permutation importance analysis revealed that a small subset of directed connectivity features accounted for most of the classification performance. The top five features had importance values ranging from 0.035 to 0.044 (mean decrease in accuracy). This suggests that this subset of directed connections strongly contributed to distinguishing prodromal from manifest PD.

Supplementary Table S2.

| No. | Feature Name (directed connection) | Importance (Mean Decrease in Accuracy) |
| --- | --- | --- |
| 1 | Default to visual network | 0.0438 |
| 2 | Visual to somatomotor network | 0.0394 |
| 3 | Control to limbic network | 0.0372 |
| 4 | Default to dorsal attention network | 0.0364 |
| 5 | Somatomotor to dorsal attention network | 0.0350 |
